# Effect of Sulfur Content in Sulfate-Rich Copper Tailings on the Properties of MgO-Activated Slag Materials

**DOI:** 10.3390/ma15124340

**Published:** 2022-06-20

**Authors:** Peiyuan Chen, Fan Yang, Xin Qian, Yi Fang, Jin Li, Xueyan Chen, Yonghui Wang

**Affiliations:** 1 School of Civil Engineering and Architecture, Anhui University of Science and Technology, Huainan 232001, China; pychen@aust.edu.cn (P.C.); jinli@aust.edu.cn (J.L.); 2019200295@aust.edu.cn (X.C.); 2021100042@aust.edu.cn (Y.W.); 2 Department of Civil and Environmental Engineering, The Hong Kong Polytechnic University, Kowloon, Hong Kong; yfang20@crimson.ua.edu; 3 Key Laboratory of Infrastructure Durability and Operation Safety, Airfield of CAAC, Tongji University, Shanghai 201804, China; 1910919@tongji.edu.cn

**Keywords:** sulfate-rich copper tailings, MgO-activated slag materials, compressive strength, hydration products

## Abstract

The high-value utilization of sulfate-rich tailings (SRCTs) can accelerate their mass consumption, so the many problems caused by the massive accumulation of SRCTs can be alleviated, such as environmental pollution, land occupation, security risk, etc. This study proposes using SRCTs to replace fine natural aggregates in MgO-activated slag materials (MASMs) and investigate the influence of the sulfur content in SRCTs on the properties of MASMs. The experimental results showed that the 28 d compressive strength of MASM mortars was increased by up to 83% using SRCT composites. Two major mechanisms were discovered: additional hydration product formation and pore structure refinement. The results of XRD suggested that incorporating SRCT composite into MASMs increased the production of expansive sulfate-containing hydration products, such as ettringite, gypsum, and hydroxyl-Afm. The results of element mapping showed that the oxidation of pyrite in SRCTs could release sulfates into the surrounding area and participate in the hydration of MASM, indicating that SRCTs can work as an auxiliary activator for MASMs. Furthermore, the addition of SRCT significantly refined the pore structure of MASMs, leading to the reduction in porosity by up to 37.77%. These findings confirm a synergistic effect on activating the slag between SRCTs and MgO, promoting the mass utilization of SRCTs. As a result, the additional expansive hydration products contribute to the enhanced compressive strength and refined pore structure.

## 1. Introduction

The copper mining industry supplies the world’s demand for copper metal while producing enormous amounts of waste. Because copper ores only contain a tiny amount of copper, 0.87% in most Chinese copper ores [1], most copper ores become wastes after mining. Onuaguluchi and Eren reported that 1 ton of copper production produces about 128 tons of copper tailings [2]. To date, most copper tailings are directly discharged into tailing ponds without effective utilization [3]. There are emergency requirements on the disposal and utilization of copper tailings because they not only pose environmental risks, but also present security issues directly related to human health.

However, sulfides are often associated with copper ores. Some of the copper tailings are sulfate-rich copper tailings (SRCTs) with excessive sulfur contents. Pyrite is one of the most common sulfides in SRCTs. It is reactive and can be easily oxidized to sulfate ions, resulting in acid mine drainage (AMD) [4] through a general reaction as expressed in Equation (1) [5,6,7],

4FeS_2_ + 15O_2_ + 8H_2_O→2Fe_2_O_3_ + 8SO_4_^2−^ + 16H (1)

The oxidation nature of SRCTs makes them challenging to bond with Portland cement. The released H^+^ reacts with the produced Ca(OH)_2_ from cement hydration, reducing the pH of pore solutions. Because the acidic environment reduces the stability of C-S-H gel, more C-S-H gel is decalcified by sulfate ions, forming gypsum in the gel pores [8,9]. Swelling secondary gypsum and highly expansive ettringite are produced from the reaction between Ca(OH)_2_ and the generated SO_4_^2−^, leading to excessive internal expansion stress and high risks of cracking of cement-based materials. For instance, Yin et al. [10] reported that there is a sub-linear and proportional relationship between expansion ratio and sulfur content in SRCTs. When the sulfur content in SRCTs is more than 8%, the formation of expansive minerals can eventually result in a partial or complete collapse of the produced cemented tailings backfill (CTB). Dong et al. [11] found that the 90 d strength of all the CTB specimens decreased under the influence of sulfide in SRCTs when using cement as the binder, and the strength of the CTB specimen with the highest sulfur content decreased the most. Solving the incompatibility problem of SRCTs with cement, especially sulfate resistance, is one of the pivotal issues regarding utilizing SRCTs.

Numerous studies have shown that alkali-activated slag materials (AASMs) possess better sulfate resistance than cement-based binders [12,13,14]. The basic logic of those findings is that the stability of hydration products of AASMs can be sustained because there is little Ca(OH)_2_ and no monosulfate available, which are susceptible substances to sulfate attack in cement-based materials [15]. Dener et al. [16] confirmed that AASMs are more resistant to Na_2_SO_4_ attack than cement-based materials. Moreover, Zhao et al. [17] claimed that AASMs subjected to 5% Na_2_SO_4_ underwent further hydration with the formation of additional ettringite. Komljenović et al. [18] stated that the hydration products of AASMs, such as C-(A)-S-H and hydrotalcite gels, exhibit high resistance to sulfate attack. Those findings suggested that AASMs are promising binders for SRCTs.

However, in AASM systems, traditional activators such as alkali-metal hydroxide and silicates are limited in practical applications because of a few obstacles, such as over-rapid setting, risk of handling caustic alkalis, and low cost efficiency [19]. In addition, the use of strong alkalis, such as NaOH and KOH, can introduce excessive drying shrinkage [20], which has detrimental effects on the stability of AASMs. In recent years, reactive MgO has emerged as a viable alternative, providing effective, economical, and environmental advantages compared with strong alkalis activators [9,19]. Shen et al. reported that reactive MgO can reduce the shrinkage ratio of AASMs due to the swelling effect of MgO [21]. Reactive MgO is also a weak alkali and can avoid harmful risks during operation [22]. When using reactive MgO as an activator, the hydrolysis of MgO can generate alkalis to promote the breaking of covalent bonds in slag to produce hydration products such as M-S-H, C-(A)-S-H, and hydrotalcite (Ht) [19]. The performance of produced MgO-activated slag materials (MASM) can be increased with a higher dosage of MgO [23]. Moreover, previous studies have confirmed that MASMs are a proper binder for SRCT-based CTB [9]. 

However, the effect of sulfur content in SRCTs on the hydration process or mechanical properties of MASMs remains unknown. Previous studies mostly focused on preparing SRCT-based CTB using MASMs, with limited reports on MASM mortars. Another significance of binding SRCTs using MASMs is that the sulfates generated through pyrite oxidation can be a potential activator. In other words, SRCT is reactive and may work as an auxiliary activator in addition to MgO. Therefore, a synergetic activating effect may be achieved by combining MgO and SRCTs [24]. Hence, it can be anticipated that SRCTs can co-activate slag with MgO, leading to significant strength enhancements. To this end, an experimental program was carried out to examine the effect of sulfur content of SRCTs on the performance of MASMs. The underlying mechanisms were analyzed using X-ray powder diffraction analysis (XRD), scanning electron microscopy (SEM), and mercury intrusion porosimetry (MIP) test. 

## 2. Materials and Methods

### 2.1. Materials

**Figure 1:**
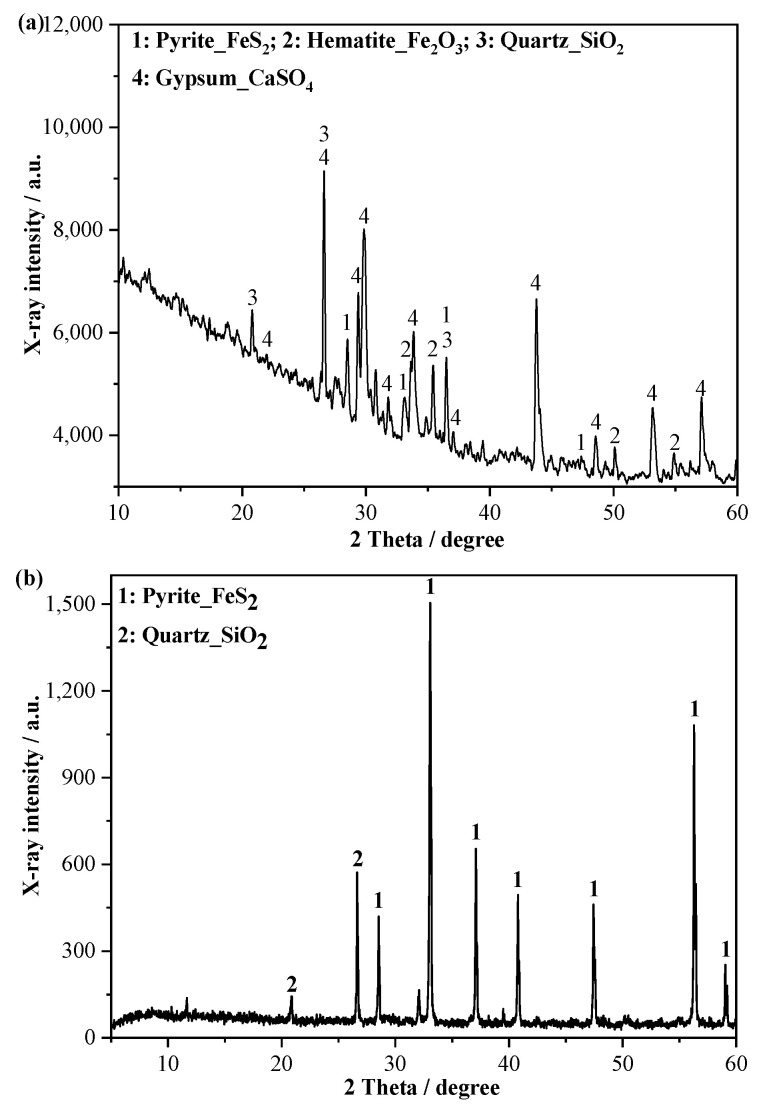
XRD patterns of the SRCTs (**a**) and pyrite (**b**) [26].

SRCTs (200 mesh) with a mean size of 76.0 μm was obtained from a local copper mine in Tongling, China. Grade S105 blast furnace slag determined by GBT18046 [25] was used with an average particle size of 17.82 μm and an apparent density of 2800 kg/m^3^. Fine pyrite was purchased to adjust the sulfur content of SRCT from a local mining company. Figure 1 presents the XRD patterns of the SRCT and pyrite [26]. As shown in Figure 1a, SRCT contains four minerals: pyrite (FeS_2_), gypsum (CaSO_4_), quartz (SiO_2_), and hematite (Fe_2_O_3_). The existence of gypsum suggests that some pyrite in SRCTs was already oxidized to produce sulfate in the natural environment. The chemical compositions of SRCTs, slag, and fine pyrite were measured by X-ray fluorescence (XRF), as shown in Table 1. Reactive MgO was chosen as the activator with a purity higher than 96%. Quartz sand powder was used. Additionally, the SRCTs, pyrite, and quartz sand possessed similar size distributions.

### 2.2. MASM Sample Preparation

In this study, the sulfur content of SRCTs was explored as the experimental variable to investigate its influence on the properties of MASMs. In this regard, quartz sand, SRCT, and fine pyrite were mixed to prepare six SRCT composites with sulfur contents of 0%, 5%, 10%, 13%, 15%, and 20% (S, wt.%). Those SRCT composites were mixed based on the sulfur contents of quartz sand, SRCTs, and fine pyrite, according to Table 1. Then, those SRCT composites were used as the total replacement of the quartz sand in MASMs. Table 2 shows the mixing proportions of MASMs with different SRCT composites. In this table, M0S represents the control group produced without SRCTs with a water-to-binder ratio of 0.6. Group M*y*S represents the MASM mortars made with SRCT composite with *y*% sulfur, whereas M13S represents MASM mortars with SRCT only. All mortars were cast into 50 × 50 × 50 mm^3^ cubic samples for compressive testing. The produced samples were cured at 23 °C with relative humidity higher than 95% until the test day.

### 2.3. Test and Characterization

#### 2.3.1. Compressive Strength

The compressive strengths of the produced mortar samples were measured by compressive strength testing using a DYE-300 machine (Wuxi Oke Electronics Co., Ltd., Jiangsu, China) with a loading rate of 1 kN/s according to GB/T 11837 [27]. Three duplicated samples were tested at the ages of 3d, 7d, and 28d. The average value was taken as the compressive strength value of this group at each date.

#### 2.3.2. X-ray Powder Diffraction (XRD)

XRD was used to evaluate the effect of SRCTs on the mineral composition of hydration products in produced MASMs. The samples were scanned in two modes, coarse scanning and fine scanning modes, using a Smartlab SE X-ray diffractometer with a Cu source (40 kV, 50 mA) [28]. For the coarse scanning mode, the scanning scope and scanning speed were 5° to 70° and 5°/min, respectively. For the fine scanning mode, the scanning scope and scanning speed were 8° to 13° and 0.5°/min, respectively. At 7 and 28 d, all the specimens were crushed in a sealed bag and then soaked in anhydrous ethanol to entirely stop the reaction. After 7 d soaking, the samples were taken out and dried in a vacuum chamber. Then, the specimens were crushed and ground into fine powders, and the particles sized less than 0.15 mm were sieved out for examination.

#### 2.3.3. Scanning Electron Microscopy (SEM)

A FlexSEM 1000 SEM with its energy-dispersed X-ray spectroscopy (EDS) (HITACHI, Japan) system was used to investigate the effect of SRCTs on the microstructure, morphology, and element migration of the specimens. Similar to specimens prepared for XRD analysis, all the samples were soaked in anhydrous ethanol and vacuum dried. To obtain high-quality backscattered electrons (BSE) images, the freshly cut and hardened mortars were cast into the capsule filled with epoxy resin, followed by being polished successively with SiC sanding paper at 400, 800, 1200, and 2000 grit for 2 min each. All the samples were coated with platinum (Pt) particles to improve electrical conductivity.

#### 2.3.4. Mercury Intrusion Porosimetry (MIP)

MIP was carried out to investigate whether SRCTs can change the pore structure of the produced mortars. The crushed samples after the compressive strength test at the age of 28d were used in the MIP analysis with a Micromeritics AutoPore IV 9510. All the specimens were vacuum-dried to constant weight prior to testing.

## 3. Results and Discussion

### 3.1. Mineral Compositions of MASM

**Figure 2:**
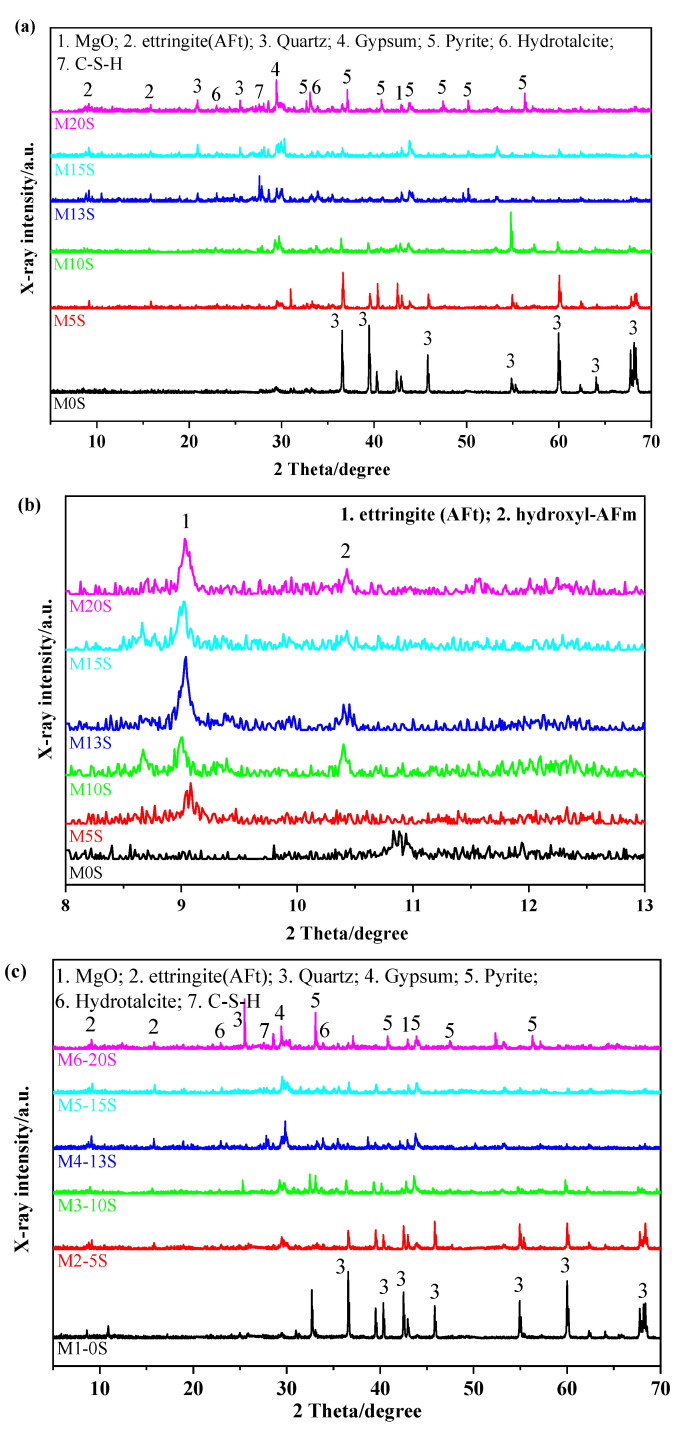

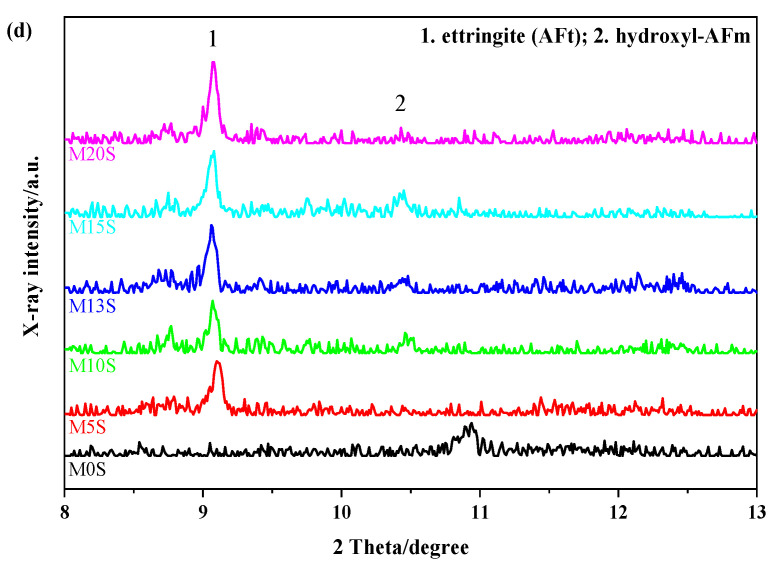
XRD patterns of MASMs with and without SRCT composite using both coarse and fine scanning modes: (**a**,**b**) 7d; (**c**,**d**) 28d.

XRD analysis was carried out to investigate the mineral compositions of the mortars prepared with different SRCT composites at 7 and 28d, and the results are presented in Figure 2. As shown in Figure 2a, for M0S without SRCTs, most of its phases in both 7 and 28d were similar to quartz as its major composition along with tiny unreacted MgO. The incorporation of SRCT composites greatly changed the mineral compositions of the MASMs. By increasing the sulfur content of the SRCT composite, there were more sulfate-containing hydration products such as gypsum and ettringite (AFt) in the SRCT-added MASMs. 

Figure 2b further presents the XRD patterns using fine scanning mode. This figure shows more details on the phase assembly of MASMs induced by the incorporation of SRCT composite. As shown in this figure, by incorporating SRCT composite, both AFt and hydroxyl-AFm were discovered in the MASMs. A higher sulfur content of SRCT composite led to a higher peak height of X-ray intensity. The above results reveal that the SRCT composite influenced the hydration properties of the MASMs through the production of sulfate-containing hydration products. The possible mechanism can be understood in that pyrite in both SRCTs and the fine pyrite were oxidized. Through that process, pyrite was converted into sulfates following the general reaction as expressed in Equation (1). The generated sulfates react with the dissolved Al-O tetrahedrons and Ca^2+^ from slag. Expansive hydration products such as gypsum and ettringite can be produced, as expressed by Equations (2) and (3) [9]. Hence, the concentrations of Ca^2+^ in the pore solutions of AASMs are reduced, which in turn accelerates the dissolution of slag to release more Ca^2+^ into the pore solutions until the ionic equilibrium can be reached. Therefore, SRCTs may work as an auxiliary activator to speed up the dissolution of slag and participate in the hydration formation of slag to produce the above-found sulfate-containing hydration products. From this point of view, SRCT is substantially reactive and thus can be an auxiliary activator in addition to MgO for AASMs.

SO_4_^2−^ + Ca^2+^ +2H_2_O→CaSO_4_·2H_2_O (2)

6Ca^2+^ +2Al(OH)_4_^−^ + 3SO_4_^2−^ + 4OH^−^ + 26H_2_O→Ca_6_Al_2_(SO_4_)_3_(OH)_12_·26H_2_O (3)

Figure 2c,d presents a similar trend in forming sulfate-containing hydration products in the mortars with SRCTs. The presence of MgO suggests the hydrolysis of MgO was still not complete after 28d of curing, as shown in Figure 2c. Less MgO was found in the mortars prepared with higher sulfur contents. This is because the hydration of slag could have been accelerated by the synergistic effect between the generated sulfates through the oxidization of pyrite and MgO, which in turn resulted in more consumption of MgO. In addition, M-S-H gel was not observed in these spectra due to its poor crystallinity [29]. 

### 3.2. Microstructure Analysis of MASM Mortars

**Figure 3:**
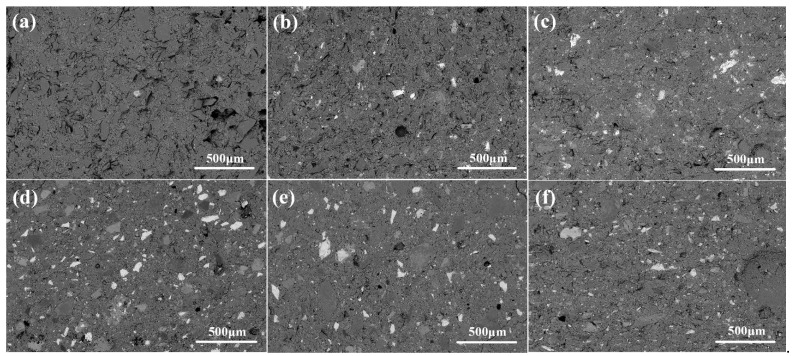
BSE images of 28 d MASM mortars: (**a**) M0S; (**b**) M5S; (**c**) M10S; (**d**) M13S; (**e**) M15S; (**f**) M20S.

Figure 3 shows the BSE images of M0S and M13S as typical samples used to investigate the distribution as well as the overall interface of SRCTs. Because BSE images depend on the average atomic number of the polished samples, the grey levels in these images correspond to SRCTs, un-hydrated slag, hydration products, and pores from light to dark, as shown in Figure 3d. SRCTs were in a well-dispersed system in the microstructure of the MASMs. The interfacial transition zone between slag and SRCTs presented a state of continuous hydration as no clear gap between SRCTs and slag was found. We attributed this finding to the sulfate released from the oxidation of SRCTs, which can accelerate the hydration of slag or form ettringite, thus contributing to an enhanced interface.

**Figure 4:**
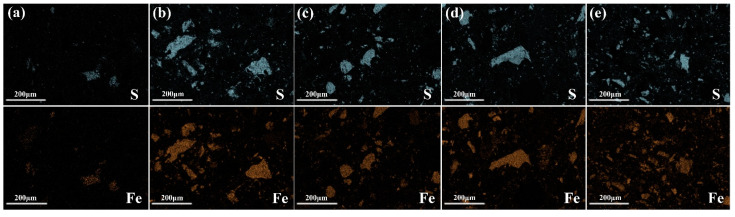
Element mapping images of 28d MASMs with different SRCT composites: (**a**) M5S; (**b**) M10S; (**c**) M13S; (**d**) M15S; (**e**) M20S.

Because sulfide mainly exists in the form of pyrite in SRCTs, most sulfur should co-exist with iron in the AASM mortars at the beginning of hydration. Therefore, the migration of sulfur can be obtained by comparing the element distribution of iron and sulfur. To this end, element mapping was employed to investigate the sulfur released from pyrite, as shown in Figure 4. It can be clearly seen that the area corresponding to S was reduced compared to that of Fe. This reduction indicates that the sulfur in the pyrite migrated into the surrounding paste. To further depict this migration feature of sulfur from pyrite, the migration rate of S in the pyrite was investigated using Image J software (Version 1.8.0, developed by National Institutes of Health, Bethesda, MD, USA) by calculating the area corresponding to the sulfur and iron in Figure 4, and the results are shown in Table 3. Similar to the increase in the sulfur content of the SRCT composite, the migration rate of S in the pyrite continuously increased from 4.23% of M5S to 17.21% of M20S. Although the calculated value of migration rate cannot accurately represent the actual migration of S, it was still indicated that S in the form of pyrite underwent oxidization, leading to the generation of sulfates. Thus, the sulfates participated in the hydration process of slag, driving its migration into the surrounding paste. Earlier XRD results also confirmed the formation of sulfate-containing hydration products due to the incorporation of SRCTs into MASMs.

**Figure 5:**
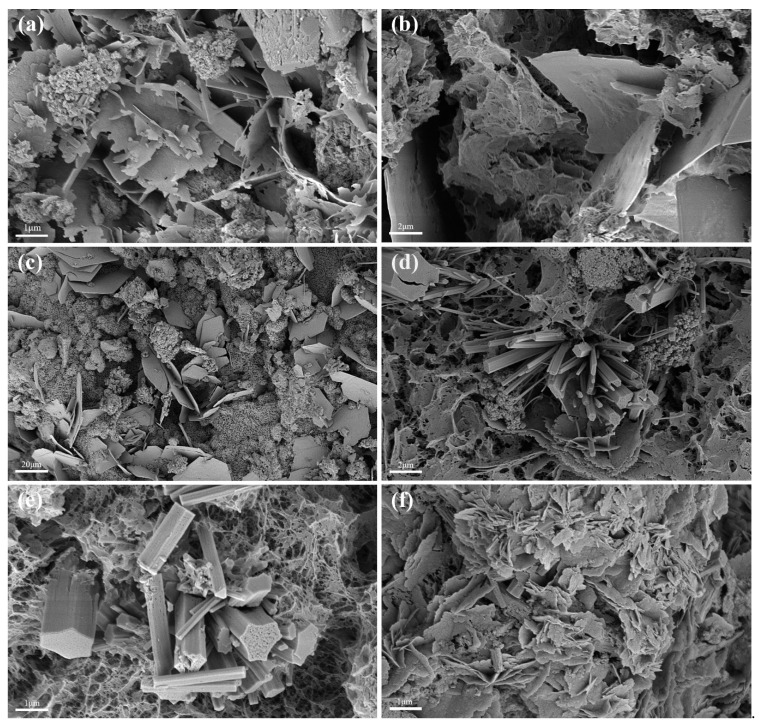
SEM images of 28d MASMs: (**a**) M0S; (**b**) M5S; (**c**) M10S; (**d**) M13S; (**e**) M15S; (**f**) M20S.

Figure 5 shows representative SEM images of 28d MASMs with different SRCT composites. It can be seen that many lamellar crystals were found in mortars without SRCTs, which were likely hydrotalcite [30]. A mesh gel structure was obtained when SRCT composite with 5% sulfur content was added, as shown in Figure 5b, suggesting a higher degree of hydration. This was mainly attributed to the auxiliary activating effect of the generated sulfates on the hydration of MASM. Figure 5d,e shows some rod-like products, possibly ettringite and gypsum, according to the results of XRD analysis. Based on the above analysis, the auxiliary activation effect exerted by the generated sulfates through the oxidization of pyrite can be confirmed, further influencing the compressive strength and pore structure of MASMs.

### 3.3. Compressive Strength of MASM Mortars

**Figure 6:**
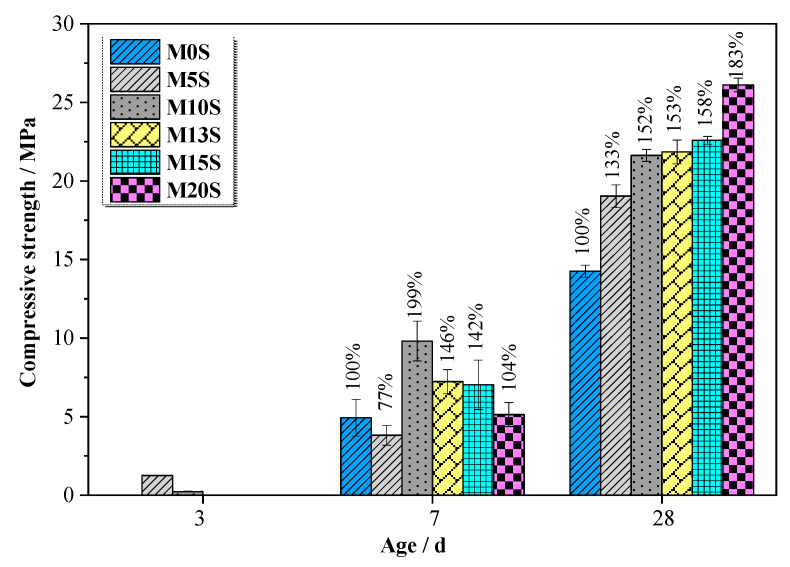
Compressive strength of MASMs.

Figure 6 presents the compressive strength of MASM mortars at 3d, 7d, and 28d. The strength development of all MASMs was extremely slow at an early age. This is because MgO is a comparatively weak alkali compared with NaOH and KOH. The hydrolysis of MgO cannot provide a sufficient alkaline environment to break the covalent bonds (Si–O–Si and Al–O–Si) in the slag, so that few hydration products can be formed at an early age [31]. At 7d, most mortars prepared with SRCTs possessed higher compressive strength than those prepared with pure quartz sand, except M5S. An almost 100% strength enhancement was obtained in the mortar prepared with SRCTs with a 10% sulfur content. This is because sulfate can also be generated from the oxidation of pyrite in the alkaline solution [32]. As mentioned earlier, sulfate can activate the slag along with MgO, thus resulting in a much higher compressive strength. Furthermore, there is a synergistic effect between sulfate and MgO on enhancing the performance of AASMs [24]. The presence of sulfate can activate the slag along with the MgO at the same time. 

However, the oxidation of pyrite can consume some of the hydroxide released from the hydrolysis of MgO, causing a delay in the hydration of slag. When the strength provided by the auxiliary activation is less than the strength loss due to the delayed hydration, the strength development of produced mortar can be even slower than the control group, as found in the case of the SRCT composite with a 5% sulfur content. This strength enhancement continues to grow with a longer curing time. The compressive strength of the mortars prepared with SRCT composite with 5%, 10%, 13%, 15%, and 20% sulfur content was higher than the control group by 33%, 52%, 53%, 58%, and 83% at the age of 28d, respectively. 

Overall, the improvement in the compressive strength is caused by two primary working mechanisms based on the above analysis: (1) the addition of SRCTs tunes the mineralogy of the hydrated AASMs by producing more expansive sulfate-containing products, such as ettringite and gypsum; (2) the hydration of slag is accelerated due to the synergistic effect between MgO and sulfate. Because of these two mechanisms, a much denser microstructure can be formed in the mortars prepared with SRCTs compared with those prepared with quartz sand. As a result, the compressive strength of the mortar using SRCTs is much higher than that using natural sand. 

### 3.4. Pore Structure of MASM Mortars

**Figure 7:**
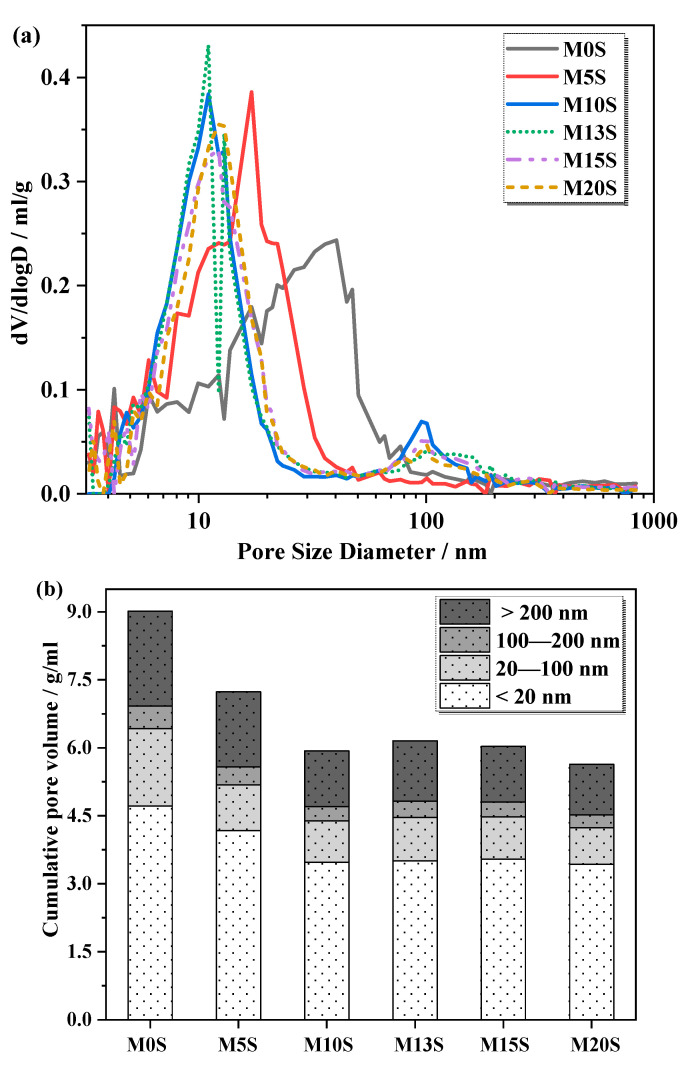
Pore structure of 28d MASM mortars: (**a**) differential pore-size distribution; (**b**) cumulative porosity.

Figure 7 shows the MIP results of 28d MASM mortars with and without SRCT composite. As shown in Figure 7a, the MIP curves of SRCT-incorporated mortars are generally on the left side of that of M0S. In addition, the major peaks of the MIP curves tend to shift to the left side of this figure when SRCT composites contain more sulfur contents. This change in the MIP curves among MASM mortars indicates that the incorporation of SRCT composite into MASM modified its pore structure by forming a denser microstructure. Figure 7b further presents the calculated accumulative pore volumes within different zones based on Figure 7a. The total porosity of 28d AASM mortars reduced from 9% to 7.2% by replacing partial natural sand with SRCT composite. When SRCT composite replaced all the quartz sand, the porosity was further reduced to 5.6% (M13S). The amount of harmful pores (>200 nm) [33] was significantly reduced, which is often observed in MASMs with a higher degree of hydration [22]. All these findings agree well with the above results. Thus, the hypothesis regarding the auxiliary activation of SRCTs is further confirmed because the introduction of SRCTs into the MASMs additionally produced expansive sulfate-containing products into the microstructure of MASMs. As a result, refined pore structure can be achieved in SRCT-incorporated MASM mortars.

## 4. Conclusions

This study investigated the influence of the sulfur content in SRCTs on the properties of MASMs. The related working mechanisms of SRCTs on the compressive strength and pore structure of MASMs were studied. The main conclusions made in this study are summarized below:

- The incorporation of SRCT composite into MASM resulted in the additional production of expansive sulfate-containing products. Those sulfate-containing products were ettringite, gypsum, and hydroxyl-AFm. The original sulfates participating in the hydration of slag were a result of the oxidation of the pyrite in SRCTs. From this point of view, SRCTs substantially worked as an auxiliary activator for the MASMs.
- MASM mortars with SRCT composite possessed much higher compressive strengths. The higher sulfur content of SRCTs ensured a higher compressive strength of the MASMs. The 28d compressive strength of MASM increased by 33% to 83% using SRCT composites with sulfur contents of 5% to 20%.
- The pore structure of MASM was also significantly refined by the incorporation of SRCT composite. The major peaks of the MIP curves tended to shift to the side of smaller pore sizes when SRCT composite had higher sulfur contents. When SRCT composite replaced all the quartz sand, the total porosity further reduced from 9% to 5.6%. The refined microstructure was attributed to the additional formation of expansive sulfate-containing products induced by the incorporation of SRCT.

Future studies should consider the shrinkage and durability of SRCT-based construction materials.

## Figures and Tables

**Table 1:** Chemical compositions of SRCTs, slag, and fine pyrite (%).

Oxide Composition	CaO	MgO	Al_2_O_3_	SiO_2_	SO_3_	Fe_2_O_3_
SRCTs	10.30	4.95	5.16	22.05	21.1	30.53
Slag	42.84	7.81	15.37	26.49	-	0.33
Fine pyrite	0.70	0.30	2.10	8.30	47.30	41.40

**Table 2:** Mix proportions of MASMs (g/100 g).

Group	MgO	Slag	Water	Quartz Sand	SRCT	Fine Pyrite	Sulfur Content
M0S	3.125	28.125	18.75	50.00	0	0	0%
M5S	3.125	28.125	18.75	30.77	19.23	0	5%
M10S	3.125	28.125	18.75	11.54	38.46	0	10%
M13S	3.125	28.125	18.75	0	50.00	0	13%
M15S	3.125	28.125	18.75	0	2.5	47.5	15%
M20S	3.125	28.125	18.75	0	8.75	41.25	20%

**Table 3:** Migration rate of sulfur of the 28d MASM based on Figure 4.

Group	5%S	10%S	13%S	15%S	20%S
Migration rate	4.23%	8.31%	11.15%	14.43%	17.21%

## Data Availability

Data is available on request.
